# 

**Published:** 1891-05

**Authors:** 


### BOOKS AND PAMPHLETS RECEIVED.

Original Research in relation to Annual Œconomics. A Socialistic study by Frank S. Billings, M. D. Rep. from Times and Register. 1891.

Annual Report of the Presbyterian Eye, Ear and Throat, Charity Hospital. Baltimore, 1891.

Uber Hufkrankheiten und ihre Behandling von Prof. E. Hess. Thiermedezinesche Vortrage. Bd. 111, Heft 3-6., herausgegeben von Dr. Georg Schneidemuhl. Leipzig Arthur Felix. February 1891.

Verhandlungen des naturhistorischen vereines der preussischen Rheinlande, westfalens und des Reg. Bezirks Osnabruck Bonn. 1890.

Verhandlungen der K. K. Zoologisch-botanischen Gesellschaft in Wein. Bd. XL. Q. 3-4. 1890.

Bericht des Naturwissenschaftlichen Vereins in Augsburg. 1890.

Mittheilungen des ornithologischen Vereines in Wein. 1891.

Bulletin de la Societé Zoologique de France. Paris, 1891.

Bulletin de l'Academie Royal de Medicine de Belgique. T. V. No.2 .

Anales de la Sociedad Cientifica Argentina, Buenos Aires, 1891.

